# Experimental and computational analysis of the secretome of the hyperthermophilic archaeon *Pyrococcus furiosus*

**DOI:** 10.1007/s00792-013-0574-0

**Published:** 2013-08-27

**Authors:** G. Schmid, G. Mathiesen, M. O. Arntzen, V. G. H. Eijsink, M. Thomm

**Affiliations:** 1 Lehrstuhl für Mikrobiologie, Universität Regensburg, Universitätsstr. 31, 93053 Regensburg, Germany; 2 Hyperthermics Regensburg GmbH, Josef-Engert-Straße 9, 93053 Regensburg, Germany; 3 Department of Chemistry, Biotechnology and Food Science, Norwegian University of Life Sciences, P.O. Box 5003, 1432 Aas, Norway; 4 Biotechnology Centre of Oslo, University of Oslo, 0317 Oslo, Norway

**Keywords:** *Pyrococcus*, Exoproteome, Exoenzymes, Signal peptides, Archaea, Lipoprotein

## Abstract

Although *Pyrococcus furiosus* is one of the best studied hyperthermophilic archaea, to date no experimental investigation of the extent of protein secretion has been performed. We describe experimental verification of the extracellular proteome of *P. furiosus* grown on starch. LC–MS/MS-based analysis of culture supernatants led to the identification of 58 proteins. Fifteen of these proteins had a putative N-terminal signal peptide (SP), tagging the proteins for translocation across the membrane. The detected proteins with predicted SPs and known function were almost exclusively involved in important extracellular functions, like substrate degradation or transport. Most of the 43 proteins without predicted N-terminal signal sequences are known to have intracellular functions, mainly (70 %) related to intracellular metabolism. In silico analyses indicated that the genome of *P. furiosus* encodes 145 proteins with N-terminal SPs, including 21 putative lipoproteins and 17 with a class III peptide. From these we identified 15 (10 %; 7 SPI, 3 SPIII and 5 lipoproteins) under the specific growth conditions of this study. The putative lipoprotein signal peptides have a unique sequence motif, distinct from the motifs in bacteria and other archaeal orders.

**Electronic supplementary material:**

The online version of this article (doi:10.1007/s00792-013-0574-0) contains supplementary material, which is available to authorized users.

## Introduction

*Pyrococcus furiosus* is a heterotrophic, anaerobic, hyperthermophilic archaeon, belonging to the order *Thermococcales*. This deep branching organism was isolated from geothermally heated marine sediments near Vulcano Island, Italy (Fiala and Stetter 1986). The microbe can utilize starch and a range of other glucans, as well as peptides as carbon and energy sources. *P. furiosus* produces organic acids, H_2_ and CO_2_ as fermentation end products and is a model organism for analysis of the biochemistry and molecular biology of archaea. Recent advances in the development of genetic tools (Waege et al. 2010; Lipscomb et al. 2011) have further facilitated research on this organism and led to a stronger focus on the possible exploitation of *P. furiosus* in biotechnological applications, e.g., for biofuel production (Basen et al. 2012).

Secreted and surface-located proteins sense the environment and support both protection against toxic components and passage of nutrients into the cells. In eukarya, bacteria and archaea most proteins are translocated across the cytoplasmic membrane using the Sec pathway (Driessen et al. 1998; Pohlschroder et al. 2005). Some of these secreted proteins are retained at the surface, while others are released to the surroundings. Secretion is directed by a N-terminal signal peptide (SP) that has a positively charged N-terminus (n-region), a hydrophobic (h-) region, and a c-region containing mostly small, uncharged residues and a characteristic cleavage site (von Heijne 1990). During or shortly after translocation across the membrane, the SP is cleaved off by a signal peptidase. Depending on the type of signal peptide (primarily defined by the character of the c-region), proteins are either recognized by a signal peptidase I (SPase I) or a signal peptidase II (SPase II). SPase I substrates are often released as soluble proteins, while SPase II substrates get attached to the cell membrane by a lipid anchor (lipoproteins). The SPs of bacterial lipoproteins contain a typical lipobox with a conserved cysteine as the first residue downstream of the cleavage site to which the lipo-anchor is attached (Hayashi and Wu 1990). In archaea, no SPase II homolog has been identified to date, although many archaeal genomes, including the *P. furiosus* genome, encode for proteins with lipobox containing N-terminal signal peptides (Saleh et al. 2010). Archaea contain a third type of secretion signal referred to as SPIII signal peptides, which are similar to bacterial type IV prepilin signal peptides (Ng et al. 2009). SPIII signal peptides lack the c-region; the cleavage site occurs directly after the n-region and the h-region is left as a part of the mature protein. Another common type of secretion mechanism is the twin-arginine translocation (Tat) pathway, which allows the secretion of proteins in a folded state (Sargent et al. 1998). The extent of Tat utilization varies widely in different organisms and in many archaea no or only few potential Tat substrates have been identified in the genome (Dilks et al. 2003). It is reported that the genome of *P. furiosus* encodes for three proteins with an N-terminus similar to known Tat substrates (Dilks et al. 2003), but as no homologs to the Tat transportation system have been identified, it is doubtful that *P. furiosus* uses this pathway.

There is a lack of experimentally verified data for the composition of archaeal secretomes, and current knowledge about this topic is therefore primarily based on the results generated by predictive programs like SignalP or ExProt (Bardy et al. 2003; Saleh et al. 2010). A drawback of these commonly used programs is that they are based on sequence information of bacteria or eukaryotes. The reliability of the predictions is therefore questionable. In 2009, the first prediction program (PRED-SIGNAL) was presented, which is based on 69 experimentally verified archaeal SPs (Bagos et al. 2009). The signal peptides used as input for developing PRED-SIGNAL are derived from different orders; they include 19 SPs from the order *Thermococcales*, thereof six from *P. furiosus*.

In the present study, we describe a first study of the secretome of *P. furiosus* DSM 3638 by experimental proteomics in combination with in silico analysis. Extracellular proteins were concentrated from the supernatants of cultures grown on starch. Proteins were analyzed after in-gel trypsination using nano online liquid chromatography (nanoLC) combined with high-resolution tandem mass spectrometry (MS/MS). Further, the SP-dependent secretome of *P. furiosus* was predicted by an in silico analysis of the genome and characteristic features of the signal peptide sequences for secreted proteins and lipoproteins were identified.

## Materials and methods

### Culture medium and growth conditions

*Pyrococcus furiosus* DSM 3638 was cultivated under anaerobic conditions in sulfur-free medium based on 1/2SME medium (Fiala and Stetter 1986). The medium was supplemented with 0.1 % (w/v) starch as primary carbon source and 0.05 % (w/v) yeast extract. The medium was prepared anaerobically and reduced with 0.03 g l^−1^ Na_2_S·3H_2_O. The cultures were grown in 1-l bottles with 330 ml medium, incubated at 95 °C with shaking at 200 rpm. The growth media were inoculated with 1.65 ml (0.5 %) of a fresh *P. furiosus* pre-culture (grown until late exponential growth phase).

### Preparation of extracellular proteins

The *P. furiosus* cultures were sampled at late exponential growth phase (1–2 × 10^8^ cells/ml). The cultures were transferred to 450-ml centrifuge beakers inside an anaerobic chamber and centrifuged anaerobically for 25 min at 6,000*g*. After the harvesting the supernatants were sterile filtered (0.2 μm pore size). The proteins in the cell-free supernatant fractions were concentrated by a two-step procedure. First, the proteins of 330-ml supernatant were concentrated using a Vivacell 250 ultrafiltration unit (Sartorius AG, Germany, filter cut off 5 kDa) following the procedure provided by the manufacturer, to a volume of 20 ml, diluted with 180 ml 50 mM Tris/HCl pH 7.7 and finally re-concentrated to 20 ml. In the second step, the proteins were further concentrated to 500–700 μl using a Vivaspin 20 centrifugal concentrator (Sartorius AG, Germany, filter cut off 5 kDa). After the final concentration step, the proteins were precipitated by acetone, a step that was crucial to obtain protein samples of sufficient quality for the subsequent analyses. In short, four volumes of ice cold acetone were added followed by 2 h incubation at −20 °C. After centrifugation (15 min, 21,500*g*, 4 °C) the supernatants were carefully discarded and the protein pellets were dried at room temperature. The protein pellets were dissolved in 100 mM Tris/Cl pH 7.7 and the protein concentration was determined by the Lowry assay (Simonian and Smith 2001).

### SDS-PAGE and in-gel trypsin digestion

To visualize and separate the proteins, samples from two *Pyrococcus* cultures were applied to two lanes of a 4–20 % Tris/Glycine Mini-Protean TGX gel (Bio-Rad, Hercules/California, USA). The gel was stained with Coomassie [0.2 % (w/v) Coomassie Brilliant Blue R250, 40 % (v/v) isopropanol, 7 % (v/v) acetic acid] and destained with distilled water. Gel lanes were sliced into 6 pieces with a scalpel and individual pieces were subjected to in-gel protein digestion with trypsin (Promega, Mannheim, Germany) following the protocol of Shevchenko et al. (2006). After trypsination, the 12 samples (six per lane, two parallel lanes) with digested proteins were individually desalted, using C_18_-StageTips (Rappsilber et al. 2003) and subsequently analyzed by nanoLC–MS/MS.

### Identification of proteins by Orbitrap-MS

Peptides were analyzed by an ESI-Orbitrap (LTQ Orbitrap XL, Thermo Scientific, Bremen, Germany) mass spectrometer coupled to an Ultimate 3000 nano-LC system (Dionex, Sunnyvale CA). For separation of peptides an Acclaim PepMap 100 column (120 mm × 75 μm) packed with 3 μm C18 particles (100 Å pore size) (Dionex) was used. A flow rate of 300 nl/min was employed with a solvent gradient of 7–35 % B in 40 min, to 50 % B in 3 min and then to 80 % B in 2 min. Solvent A was 0.1 % formic acid and solvent B was 0.1 % formic acid/90 % ACN. The mass spectrometer was operated in data-dependent mode in order to automatically switch between Orbitrap-MS and LTQ-MS/MS acquisition. Survey full scan MS spectra (from *m*/*z* 300–2,000) were acquired in the Orbitrap with the resolution *R* = 60,000 at *m*/*z* 400 (after accumulation to a target of 500,000 charges in the LTQ). The method used allowed sequential isolation of the most intense ions, up to six, depending on signal intensity, for fragmentation on the linear ion trap using collision induced dissociation (CID) at a target value of 10,000 charges. For accurate mass measurements, the lock mass option was enabled in MS mode and the polydimethylcyclosiloxane ions generated in the electrospray process from ambient air were used for internal recalibration during the analysis. Target ions already selected for MS/MS were dynamically excluded for 60 s. General mass spectrometry conditions were electrospray voltage, 1.6 kV; no sheath and auxiliary gas flow. Ion selection threshold was 5,000 counts for MS/MS and an activation *Q* value of 0.25 and activation time of 30 ms were in addition applied for MS/MS. Data were acquired using Xcalibur v2.5.5 and processed into searchable mgf-files using ProteoWizard v2.1.2708. The data were then searched against a local Fasta database of *P. furiosus* extracted from NCBI (4,867 sequences) using Mascot (Perkins et al. 1999) as search engine. Allowed variable post-translational modifications were: deamidation of glutamines and asparagines, oxidation of methionines, propionamidylation of cysteines, and, for peptides with N-terminal glutamines, conversion of glutamine to pyro-glutamic acid. As enzyme trypsin was chosen and the maximum number of allowed miscleavages was 1. The accuracy of precursor ions was set to 10 ppm and for fragment ions 0.6 Da. Mascot result files were imported into Scaffold v.3.00.08 (Proteome Software, Portland, Oregon, USA) (Searle 2010) and researched with X!Tandem with default parameters. For valid protein identification at least 1 unique peptide in both parallels was required with a probability ≥95 % and total protein probability ≥98 %. For protein quantification unique peptide counts were exported from Scaffold and emPAI values (Ishihama et al. 2005) were calculated using an in-house python-script.

### Bioinformatic analysis of identified proteins

All identified proteins in the supernatant fractions were analyzed for N-terminal signal sequences using the programs SignalP 4.0 (Petersen et al. 2011) and PRED-SIGNAL (Bagos et al. 2009). PRED-SIGNAL was used to do a genome-wide signal peptide analysis of *P. furiosus*. The genome sequence was extracted from the NCBI gene bank (http://www.ncbi.nlm.nih.gov/genome/?term=pyrococcus%20furiosus). LipoP 1.0 (Juncker et al. 2003) was used to predict lipoproteins. Selected proteins were analyzed for N-terminal transmembrane segments using the Phobius web server (Kall et al. 2007). Putative domain annotations in hypothetical proteins were done using Pfam 26.0 (Punta et al. 2012).

## Results and discussion

### Proteins identified in the supernatant

**Fig. 1:**
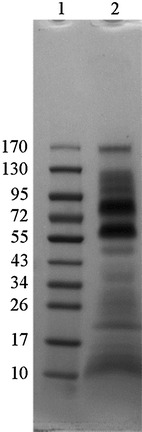
A representative coomassie stained SDS-PAGE gel showing concentrated proteins from *P. furiosus* cell-free culture supernatant. *1* PageRuler Prestained Protein Ladder; molecular weights are indicated in kDa. *2* 37 μg protein from culture supernatant

Supernatant fractions from *P. furiosus* grown on starch as major carbon source were collected in the late exponential growth phase, using anaerobic conditions during harvesting to prevent cell lysis. The proteins in the cell-free supernatant were concentrated by ultrafiltration followed by acetone precipitation. The concentrated proteins were separated by 1D-gel electrophoresis (Fig. 1) and converted to tryptic peptides by in-gel trypsination. The peptides were then analyzed using high resolution LC–MS/MS as described in the “Materials and methods” section.

Using this approach, 58 proteins were identified (Tables 1, 2), including major enzymes for starch degradation. Previous microarray analysis have shown that the amylopullulanase PF1935*, the maltotriose binding protein PF1938 and the hypothetical protein PF1109 are the only extracellular proteins that are specifically up-regulated when *P. furiosus* grows on starch (Lee et al. 2006). All these proteins were also found in the current study (Table 1), confirming their importance in starch metabolism. Although quantification of proteins is not straightforward in the present type of LC–MS/MS experiments, it is possible to obtain a relative measure of protein quantities by quantifying the number of peptide counts acquired per protein. Since this number will be biased by differences in the occurrence of basic residues (i.e., tryptic cleavage sites), the peptide counts need to be corrected by a factor representing the number of likely observable peptides for a given protein. This approach yields the so-called emPAI value (exponentially modified protein abundance index) (Ishihama et al. 2005). As expected, the emPAI quantification of the data indicated that the above-mentioned proteins involved in starch metabolism were among the most abundant in the culture medium (Table S1).

**Table 1:** Proteins with a predicted N-terminal signal sequence identified in the supernatant of *P. furiosus* DSM 3638 grown on starch

Gene	Accession number	Gene product	Pfam^a^	Predicted signal sequence	MW (kDa)	Unique peptides^b^	Total coverage (%)
PF0119	18892036	Periplasmic sugar binding protein		SP II	61.2	8	20
PF0190	18892121	Hypothetical protein	Bacterial extracellular solute-binding proteins	SP I	94.8	36	44
PF0287	18892232	Pyrolysin		SP III	154.4	28	27
PF0337	18976709	Flagellin		SP III	28.6	4	25
PF0477	2183106	Alpha amylase		SP I	52.9	9	21
PF1109/PF1110^c^	18893182 18893183	Extracellular starch binding protein		SP I		20/7^c^	28/44^c^
PF1209	18893298	Oligopeptide ABC transporter (oligopeptide-binding protein)		SP I	71.6	3	4
PF1304	18893406	Hypothetical protein	Unknown function	SP III	93.7	9	15
PF1399	18893514	Putative ATPase, vanadate-sensitive		SP I	65.7	24	52
PF1408	18893525	Putative dipeptide-binding protein		SP II^e^	79.4	30	52
PF1505	18893637	Hypothetical protein	Translocon-associated protein beta	SP I	78.5	14	25
PF1695	18893856	Hypothetical lipoprotein	Basic membrane protein	SP II	44.2	9	21
PF1774	18893949	Iron (III) ABC transporter, ATP-binding protein		SP II	40.3	11	46
PF1935*^d^	75993212	Amylopullulanase		SP I	127.1	52	47
PF1938	18202323	Maltotriose-binding protein		SP II	48.2	20	59

^a^Significant hits obtained after searches in Pfam 26.0 (Punta et al. 2012) for hypothetical proteins

^b^The column shows the cumulative number of unique peptide hits from two biological replicates. A protein was considered as significant if it was identified by at least one unique peptide in both parallels

^c^A recent study has shown, that *pf1109* and *pf1110* represent a single ORF, encoding a starch-binding protein (Comfort et al. 2008). In the text we are using the term PF1109 for this gene product. In the Table, we have split the total number of detected peptides and the total coverage into the numbers detected for each of the two originally annotated ORFs

^d^The previously annotated ORFs pf1934 and pf1935 are one continuous gene, now termed pf1935* (Lee et al. 2006)

^e^This protein was among the 18 SPIII proteins predicted by Szabó et al. (2007). We conclude that PF1408 is a lipoprotein (see text)

**Table 2:** Proteins without predicted N-terminal signal sequences identified in the supernatant of *P. furiosus* DSM 3638 grown on starch

Functional group	Gene	Accession number	Gene product (NCBI)	Pfam^a^	MW (kDa)	Unique peptides^b^	Total coverage (%)	Phobius TM domain^c^
Energy and metabolism	PF0043	18891945	Phosphoenolpyruvate synthase		90	11	17	No
	PF0289	18892234	Phosphoenolpyruvate carboxykinase		73	4	6	No
	PF0346	18892300	Aldehyde ferredoxin oxidoreductase		67	32	57	No
	PF0272	1351936	Alpha-amylase		76	10	15	No
	PF0456	18892427	Carboxypeptidase 1		59	8	12	No
	PF0588	18892584	Phospho-sugar mutase		50	7	19	No
	PF0597	18976969	IAA-amino acid hydrolase		49	4	10	No
	PF0751	18892770	Flavoprotein		47	5	14	No
	PF0825	18892854	Prolyl endopeptidase		71	6	11	No
	PF0965	1197364	Pyruvate ferredoxin oxidoreductase beta-2		36	6	28	No
	PF0966	1197363	Pyruvate ferredoxin oxidoreductase alpha-2		44	7	17	No
	PF1203	18893290	Formaldehyde ferredoxin oxidoreductase		70	18	24	No
	PF1266	18893362	Cystathionine gamma-lyase		41	8	32	No
	PF1283	18893381	Rubrerythrin		20	5	26	No
	PF1394	18893507	Phosphoglycerate dehydrogenase		34	5	16	No
	PF1421	18893540	Hypothetical 4-aminobutyrate aminotransferase		51	15	42	No
	PF1472	18893598	Aspartate/serine transaminase		43	4	14	No
	PF1480	18893608	Formaldehyde ferredoxin oxidoreductase wor5		65	3	5	No
	PF1535	18893671	Alpha-glucan phosphorylase		98	13	20	No
	PF1540	18893678	Acetyl coenzyme A synthetase		50	6	17	No
	PF1547	18893685	Endoglucanase		39	8	26	No
	PF1602	1122753	Glutamate dehydrogenase		47	12	30	No
	PF1616	18893766	Myo-inositol-1-phosphate synthase		42	8	33	No
	PF1719	1373331	Protease I		19	7	61	No
	PF1778	18893953	Serine hydroxymethyltransferase		48	11	29	No
	PF1787	18893964	Acetyl-CoA synthetase		26	8	35	No
	PF1866	18978238	S-adenosylmethionine synthetase		44	7	26	No
	PF1920	18894116	Triosephosphate isomerase		24	4	24	No
	PF1959	18894161	Phosphonopyruvate decarboxylase bcpc		45	4	11	No
	PF1961	18894163	Tungsten-containing formaldehyde ferredoxin oxidoreductase wor4		69	5	8	No
Transport	PF1933	18894131	Putative sugar transport ATP-hydrolyzing		41	5	13	No
	PF1936	18894134	Putative sugar transport inner membrane protein (malg-like)		45	4	12	6
Translation and transcription	PF1375	18893486	Translation elongation factor eF-1, subunit alpha		48	8	24	No
	PF1803	18893984	LSU ribosomal protein L30P		18	4	22	No
	PF1881	18978253	Chromatin protein		10	2	30	No
Protein folding	PF1974	18894178	Thermosome, single subunit		60	20	43	No
Cell division	PF0525	18892510	Cell division protein		44	12	33	No
Hypothetical proteins	PF0380	33359476	Hypothetical protein PF0380	ParB-like nuclease domain	28	7	25	No
	PF0547	18892536	Hypothetical protein PF0547	CobW/HypB/UreG, nucleotide-binding domain	51	13	33	No
	PF1047	18893110	Hypothetical protein PF1047	FUN14 family (unknown function)	10	2	27	3
	PF1111	18893184	Hypothetical protein PF1111	Protein of unknown function DUF43	40	5	13	No
	PF1500	18893630	Hypothetical protein PF1500	PRC-barrel domain	10	6	61	No
	PF1837	18894020	Hypothetical protein PF1837	ATP-grasp domain; Binding-protein-dependent transport system inner membrane component	26	4	19	No

^a^Significant hits obtained after search in Pfam 26.0 (Punta et al. 2012) for hypothetical proteins

^b^The column shows the number of unique peptide hits from two biological replicates. A protein was considered as significant if it was identified by at least one unique peptide in both parallels

^c^Prediction of transmembrane (TM) domains using the Phobius web server (Kall et al. 2007)

In addition to these proteins that are known to play a key role in starch degradation, we detected the α-amylase PF0477 and the periplasmic sugar binding protein PF0119. The gene *pf0477* is up-regulated when *Pyrococcus* grows on peptides, indicating that this α-amylase may be involved in a metabolic switch from peptide to α-glucan degradation, when α-glucans become available during growth on peptides (Lee et al. 2006). The sugar binding protein PF0119 is not known to be specifically expressed under glycolytic or proteolytic growth conditions and might therefore play a more general role in sugar uptake. Furthermore, we identified the serine protease pyrolysin (PF0287), which was previously shown to be cell envelope-associated (Eggen et al. 1990; Voorhorst et al. 1996), and the peptide binding proteins PF1209 and PF1408.

All the above-mentioned proteins with (putative) roles in starch and protein metabolism were among the in total 15 detected proteins in the supernatant fractions with a putative N-terminal signal peptide (Table 1, for details see below). Seven additional proteins with a predicted signal peptide were identified in the supernatant fractions, an ATPase (PF1399), an ATP-binding transporter protein (PF1774), four hypothetical proteins and a flagellin (PF0337).

In addition, we identified 43 proteins in the supernatant fraction without a typical N-terminal signal peptide (Table 2). Judged by the emPAI values, these proteins varied in abundance: some were among the most abundant of all detected proteins, whereas the majority appeared in the lower regions of the abundance list (Table S1). Most of these 43 proteins are predicted to have intracellular functions and are therefore not supposed to be actively secreted. Intracellular proteins are regularly found in the culture media of bacteria (Antelmann et al. 2001; Trost et al. 2005) and archaea (Palmieri et al. 2009; Ellen et al. 2010a) and it remains to some extent uncertain whether this is a result of artifacts such as cell lysis or whether this reflects active secretion of intracellular proteins, some of which may even have different intracellular and extracellular functions [“moonlighting” proteins; (Huberts and van der Klei 2010)]. In the case of archaea, an additional possible explanation could be protein export via membrane vesicles (Soler et al. 2008; Ellen et al. 2009, 2010b; Deatherage and Cookson 2012). Further work is needed to establish which of these possible explanations are valid.

### Signal sequences of experimentally verified extracellular proteins

The 58 proteins identified in the supernatant fractions were analyzed for N-terminal signal sequences using the programs PRED-SIGNAL (Bagos et al. 2009) and SignalP 4.0 (Petersen et al. 2011). PRED-SIGNAL is trained on signal sequences of archaea and is the only available program that specifically predicts archaeal SPs. The program predicted that 15 of the identified proteins contain N-terminal SPs (Table 1). SignalP 4.0 is the most used prediction program for SPs as it is based on a considerable number of experimentally verified eukaryotic and bacterial SPs. When the program was selected to search for signal peptides of Gram-positive bacteria, 11 of the 15 proteins selected by PRED-SIGNAL were predicted to contain N-terminal SPs. When SignalP was selected to search for eukaryotic signal peptides, 14 of the 15 proteins selected by PRED-SIGNAL were identified. The additional protein identified by PRED-SIGNAL (relative to SignalP) was a flagellin (PF0337). Previous analyses of the *P. furiosus* genome using FlaFind have led to the identification of 18 proteins putatively carrying a class III signal peptide (Szabó et al. 2007). We identified four of these proteins in the supernatant fractions (PF0287, PF0337, PF1304 and PF1408; Table 1) and all these four proteins were also predicted to be secreted by PRED-SIGNAL. For reasons described below, we conclude that one of these four, PF1408, in fact is a lipoprotein.

Analysis of the 43 proteins without a predicted signal sequence using the Phobius server (Kall et al. 2007), indicated that only two of these proteins (PF1047 and PF1963) contain transmembrane segments (3 and 6, respectively). In both these proteins one of the transmembrane segments is located N-terminally and could potentially function as a signal sequence without a cleavage site that co-directs insertion into the cell membrane.

For the detection of lipoproteins, all identified proteins were analyzed with the prediction program LipoP 1.0 (Juncker et al. 2003). Although lipoproteins are naturally attached to the cell membrane, it is not unusual to find them in the supernatant fraction as a result of natural shedding (Cole et al. 2005; Tjalsma et al. 2008; Bøhle et al. 2011). LipoP predicted that three of the 58 proteins in the supernatant fraction are lipoproteins (PF0119, PF1695 and PF1774). A manual examination of all identified proteins (see below) showed that two other proteins (PF1938 and PF1408) have features in the N-terminus very similar to the lipoproteins predicted by LipoP (Table 3). Besides a positively charged n-region and a leucine-rich h-region these proteins share the sequence G/CIGG (‘/’ indicates the cleavage site). This motif matches the lipobox motif previously suggested for *Pyrococcus* spp. (Albers et al. 2004). PF1408 has previously been predicted to harbor a class III signal peptide (Szabó et al. 2007).

**Table 3:** Predicted N-terminal signal sequences of proteins identified in the culture medium

Gene	Predicted signal peptide
SPI^a^	
PF1109	MRRNAQVFAMVLLLVLSGIPKALA/LYTPTPFSID
PF1209	MKRLVGVLIGAFVIFGVFGQVVAA/QEQELPREET
PF1399	MKVKKIAALAVGAAVAGATLGFASA/QGEVPEIPKD
PF0477	MNIKKLTPLLTLLLFFIVLASPVSAA/KYLELEEGGV
PF1935*^b^	MSRKLSLLLVFLIFGSMLGANNIVKA/EEPKPLNVII
PF0190	MRKKLVGILTILVALGMLVSPLLKPVAA/EDQKVLKIAM
PF1505	MKKASILLIIMLIASGLTIFNPKA/LGLEKYSTLT
Lipoproteins^c^	
PF1938^d^	MRRATYAFALLAILVLGVVASG/CIGGGTTTPT
PF1408^d,e^	MKKGLLAILLVGVMVLGTFGSG/CIGGGTQTQT
PF0119^c^	MKHKAVFLLVVLISGVLASG/CIGGETKETQ
PF1774^c^	MKRAIPVFLLIVLVWISG/CIGGGTSTIP
PF1695^c^	MRKVGITLSVVALVIMGFVAG/CIGGTQTQGE
SPIII^f^	
PF0337	MKKG/AIGIGTLIVFIAMVLVAAVAAGVLI
PF0287	MNKKG/LTVLFIAIMLLSVVPVHFVS
PF1304	MRRG/FIINSTLLILIIPLLLLAATYAEI

Predicted cleavage sites are indicated by “/”; the h-regions of the signal peptides are underlined

^a^Signal peptides and cleavage sites under “SPI” were predicted by the PRED-SIGNAL program that is optimized for archaea

^b^The previously annotated ORFs pf1934 and pf1935 are currently considered one continuous gene, now termed pf1935* (Lee et al. 2006)

^c^Signal peptides of lipoproteins were predicted using LipoP 1.0 combined with manual inspection of the SPI sequences (see text)

^d^Manually predicted lipoproteins (see text for details)

^e^This protein was among the 18 SPIII proteins predicted by Szabó et al. 2007. We conclude that PF1408 is a lipoprotein (see text)

^f^Signal peptides predicted previously using FlaFind by Szabó et al. 2007

**Fig. 2:**
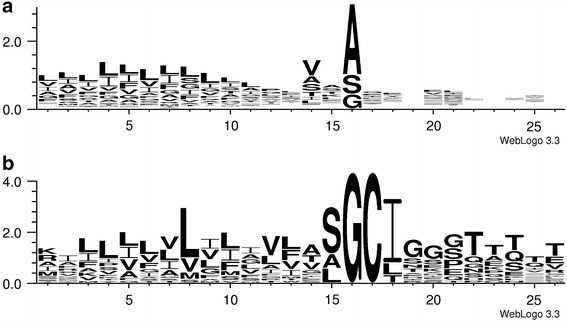
Frequency plot for signal peptides based on multiple alignment of 16 residues upstream and 10 residues downstream of predicted signal peptide cleavage sites. **a** A composition map based on 107 predicted SPase I signal sequences identified in the *P. furiosus* DSM 3638 genome. **b** A composition map based on 21 predicted lipoprotein signal sequences identified in the *P. furiosus* DSM 3638 genome. These pictures were made with WebLogo (Crooks et al. 2004)

To summarize, the 15 extracellular proteins with signal peptides detected in this study (Tables 1, 3) comprise seven proteins with a predicted SPase I cleavage site, five proteins with a putative SPase II cleavage site (lipoproteins) and three with a predicted SPase III cleavage site. The predicted SPase I signal sequences are very similar in length (24–28 amino acids) and amino acid composition (Table 3). They have two or more lysine or arginine residues at the N-terminus and a distinct hydrophobic region dominated by leucines (Fig. 2a). The signal peptides of the lipoproteins are 18–22 amino acids in length and share the motif ([S(A)]G/CIGG) around the predicted cleavage site (indicated by ‘/’) (Table 3; Fig. 2b).

### Genome-wide analysis

An analysis of the whole predicted proteome of *P. furiosus* using PRED-SIGNAL, led to identification of 166 proteins with putative N-terminal signal sequences (Table S2). Surprisingly, 31 of these proteins had an uncharged or even negatively charged N-terminus. Signal peptides usually have a positively charged N-terminus, which interacts with the negatively charged inner part of the cytoplasmic membrane (von Heijne 1990). All signal peptides from the experimentally verified signal peptide-containing secreted proteins in this study had at least two positively charged amino acids at the N-terminus (Table 3). Whether these 31 proteins without a positive net charge at the N-terminus are actively secreted remains to be seen. The SPs of the remaining 135 proteins exhibited typical key features of signal peptides (von Heijne 1990) (Table S2).

For the identification of lipoproteins we combined a computational analysis by LipoP with a manual examination. In the first step, the whole genome of *P. furiosus* was searched for lipoproteins using LipoP. The program identified 18 putative lipoproteins, two of which (PF1298, PF2063) were not identified by PRED-SIGNAL (Table S2; this brings the total number of putatively secreted proteins to 137). All 18 identified SPs contained a Gly-Cys motif at the predicted cleavage site. In a second step, all of the 135 proteins predicted to be secreted that were not predicted to be lipoproteins by LipoP were searched for the occurrence of a Gly-Cys motif within the first 25 amino acids. This analysis identified three additional putative lipoproteins, including the maltotriose-binding protein, PF1938, and the putative dipeptide binding protein, PF1408, identified in the secretome (see above and Tables 1, 3), as well as the hypothetical protein PF0978. We suggest these proteins to be lipoproteins due to their signal peptide features and the typical lipobox motif (Tables 3; S2). Six of the 21 putative lipoproteins shared the sequence SG/CIGG, which has previously been suggested to be the consensus sequence of *Pyrococcus* spp. lipoboxes (Albers et al. 2004).

Of the 18 proteins predicted by Szabó et al. (2007) to contain a class III signal peptide (Table S2), ten are predicted by PRED-SIGNAL to harbor an SP I signal peptide. One of these ten was manually predicted to be a lipoprotein (see above). This brings the total number of putatively secreted proteins to 145.

Interestingly, a frequency plot (Fig. 2b) of the 21 putative lipoproteins, including PF1408, showed that the −2 position upstream of the cleavage site is dominated by serine, representing almost two-thirds of the amino acids at that position. In bacteria the −2 position is dominated by the apolar amino acid alanine (Hayashi and Wu 1990). Another interesting feature is that the +2 position is dominated by isoleucine, with a frequency of 76 % (Fig. 2b). In bacteria isoleucine is usually not found at the +2 position (Hayashi and Wu 1990). From the +3 to the +5 position glycine is dominating, while threonine is the most abundant amino acid at the positions +6 to +10. This sequence profile can neither be found in bacteria, nor is it known from other archaea. As *Pyrococcus* is a very deep branching organism, we assume that the profile of its lipoprotein SP sequences represents an ancestral type. It is conceivable that the deviating, probably ancestral sequence profile of the lipoprotein SPs in *Pyrococcus* requires a different type of signal peptidase II, compared to the known bacterial one. This might explain why in *Pyrococcus* no signal peptidase II homolog has been identified yet.

The putative SPase I signal peptides (Table S2) were on average 5 residues longer (~25 residues) compared to the SPase II signal peptides (~20 residues), similar to what has been reported for bacteria (Klein et al. 1988; von Heijne 1989). In the n-region lysine (62 % of basic residues) is more frequent than arginine (36 % of basic residues), which reflects the common archaeal pattern (Bagos et al. 2009) (Table S2). A frequency plot for all 107 predicted SPase I signal peptides showed that the −1 position relative to the cleavage site is clearly dominated by alanine (72 SPs), while at the −3 position valine (35 SPs) is the most frequent amino acid, followed by alanine and serine (both 17 SPs) (Fig. 2a). This is in accordance with the common archaeal pattern (Bagos et al. 2009), the only exception being that, generally, the −3 residue is an alanine rather than a valine as in *P. furiosus*. Interestingly, in the h-regions of the *P. furiosus* SPs leucine is the most frequent amino acid. Such a pre-dominance of leucine in the h-region is also found in eukaryotes (Bagos et al. 2009), underpinning the possible evolutionary relationship between eukaryotes and the deep-branching archaeon *P. furiosus*.

In summary, we suggest that 145 proteins of *P. furiosus* are secreted by use of an N-terminal signal sequence, including 21 lipoproteins (Table S2). This corresponds to 6.7 % of the *P. furiosus* proteome, which is significantly less compared to a previous prediction where the secretome of *P. furiosus* was estimated to comprise 9 % of the proteome (Saleh et al. 2010). This disparity may be due to the fact that in the latter study the secretome was predicted using ExProt, a program trained on signal peptides from bacteria.

## Conclusion

Generally, little is known about the secretomes of archaea. The present study adds to a slowly growing data set which so far seems to indicate that secretion in archaea is a limited process, with only few signal-peptide containing proteins being freely secreted into the growth medium (Saunders et al. 2006; Ellen et al. 2010a). Under the specific growth conditions investigated in this study, only 15 proteins with N-terminal signal sequences were identified, that is only 10 % of all proteins with putative N-terminal signal peptides. This low number may be due to limited expression/release of secreted proteins, whereas some secreted proteins may escape detection due to them being relatively resistant to trypsination even after SDS-PAGE. The sequences of the SPase I signal peptides share features with common archaeal sequence patterns. Remarkably, the sequence motifs around the putative or predicted SPase II cleavage sites of the lipoproteins of *P. furiosus* differ from the motifs found in bacteria and other archaea. The combination of this first experimental glimpse of the secretome and our analysis of signal peptide sequences should provide a useful basis for further studies on protein secretion in this hyperthermophilic archaeon.

## Electronic supplementary material

Below is the link to the electronic supplementary material.

Supplementary material 1 (PDF 261 kb)

Supplementary material 2 (PDF 81 kb)
