# Supplementary material for: Experimental and computational analysis of the secretome of the hyperthermophilic archaeon Pyrococcus furiosus
Source: Extremophiles. 2013 Aug 27;17(6):921–30. doi: 10.1007/s00792-013-0574-0 (PMC3824201; doi:10.1007/s00792-013-0574-0)
Supplement: Supplementary file 1 — Supplementary material 1 (PDF 261 kb) [file 792_2013_574_MOESM1_ESM.pdf]

Table S1: Quantitative analysis of MS data for proteins found in the supernatant using the emPAI approach (Ishihama et al. 2005). The Table combines data from two independent experiments (SN1 & SN2)

						Unique peptides		Unweighted Spectrum Count [c]		emPAI[d]		COMBINED SN1 & SN2		
Protein	Gene	Accession Number	Secretion signal	Molecular Weight [Da]	Possible Peptides[b]	SN1	SN2	SN1	SN2	SN1	SN2	Unique peptides	emPAI[d]	Coverage
hypothetical protein PF1500	pf1500	18893630	-	10358,7	8	4	4	11	7	2,162	2,162	6	4,623	61 %
aldehyde:ferredoxin oxidoreductase (aor)	pf0346	18892300	-	66589,0	48	29	32	91	121	3,019	3,642	32	3,642	57 %
Maltotriose-binding protein	pf1938	18202323	SP II	48213,1	35	17	17	80	81	2,060	2,060	20	2,728	59 %
amylopullulanase	pf1935*	75993212	SP I	127133,4	92	50	51	423	421	2,495	2,584	52	2,675	47 %
putative dipeptide-binding protein	pf1408	18893525	SP II	79360,0	57	28	23	93	113	2,099	1,532	30	2,360	52 %
hypothetical protein PF0190	pf0190	18892121	SP I	94753,9	69	32	31	308	319	1,909	1,814	36	2,325	44 %
protease I	pf1719	1373331	-	18778,7	14	6	7	20	31	1,683	2,162	7	2,162	61 %
hypothetical protein PF1110[a]	pf1110	18893183	-	19265,4	14	7	6	25	27	2,162	1,683	7	2,162	44 %
putative ATPase, vanadate-sensitive	pf1399	18893514	SP I	65678,4	48	24	23	229	88	2,162	2,014	24	2,162	52 %
thermosome, single subunit	pf1974	18894178	-	59943,8	43	19	9	42	19	1,766	0,619	20	1,918	43 %
acetyl-CoA synthetase	pf1787	18893964	-	25861,9	19	7	3	12	4	1,336	0,438	8	1,637	35 %
hypothetical 4-aminobutyrate aminotransferase	pf1421	18893540	-	50724,5	37	15	13	38	28	1,543	1,246	15	1,543	42 %
iron (III) ABC transporter, ATP-binding protein	pf1774	18893949	SP II	40326,9	29	11	8	23	20	1,395	0,887	11	1,395	46 %
cell division protein	pf0525	18892510	-	44053,1	32	11	9	22	18	1,207	0,911	12	1,371	33 %
formaldehyde:ferredoxin oxidoreductase	pf1203	18893290	-	69634,6	50	16	11	41	19	1,089	0,660	18	1,291	24 %
rubrerythrin	pf1283	18893381	-	19487,8	14	5	4	15	13	1,276	0,931	5	1,276	26 %
glutamate dehydrogenase	pf1602	1122753	-	47084,2	34	9	12	25	25	0,840	1,254	12	1,254	30 %
hypothetical protein PF0547	pf0547	18892536	-	50724,4	37	12	5	24	14	1,110	0,365	13	1,246	33 %
hypothetical protein PF0380	pf0380	33359476	-	28019,7	20	5	3	12	4	0,778	0,413	7	1,239	25 %
serine hydroxymethyltransferase	pf1778	18893953	-	47956,4	35	8	9	17	19	0,693	0,808	11	1,062	29 %
LSU ribosomal protein L30P	pf1803	18893984	-	17698,7	13	3	3	4	5	0,701	0,701	4	1,031	22 %
endoglucanase	pf1547	18893685	-	38559,3	28	8	2	20	4	0,931	0,179	8	0,931	26 %
hypothetical lipoprotein	pf1695	18893856	SP II	44173,9	32	8	6	15	11	0,778	0,540	9	0,911	21 %
cystathionine gamma-lyase (gamma-cystathionase)	pf1266	18893362	-	41177,6	30	8	3	15	5	0,848	0,259	8	0,848	32 %
hypothetical protein PF1109[a]	pf1109	18893182	SP I	106477,1	77	20	18	224	197	0,819	0,713	20	0,819	28 %
myo-inositol-1-phosphate synthase	pf1616	18893766	-	42359,5	31	8	4	13	5	0,812	0,346	8	0,812	33 %
pyrolysin	pf0287	18892232	SP III	154378,0	111	28	21	84	74	0,788	0,546	28	0,788	27 %
hypothetical protein PF1047	pf1047	18893110	-	10477,7	8	1	2	1	6	0,334	0,778	2	0,778	27 %
chromatin protein	pf1881	18978253	-	10378,6	8	2	2	3	4	0,778	0,778	2	0,778	30 %
hypothetical protein PF1505	pf1505	18893637	SP I	78450,0	57	12	11	30	25	0,624	0,559	14	0,760	25 %
alpha amylase	pf0477	2183106	SP I	52875,8	38	8	7	16	17	0,624	0,528	9	0,725	21 %
pyruvate ferredoxin oxidoreductase beta-2	pf0965	1197364	-	36237,9	26	6	6	13	11	0,701	0,701	6	0,701	28 %
translation elongation factor eF-1, subunit alpha (tuf)	pf1375	18893486	-	47605,3	35	7	2	11	4	0,585	0,141	8	0,693	24 %
triosephosphate isomerase	pf1920	18894116	-	24051,0	18	4	3	8	4	0,668	0,468	4	0,668	24 %
pyruvate ferredoxin oxidoreductase alpha-2	pf0966	1197363	-	44158,9	32	6	4	11	7	0,540	0,334	7	0,655	17 %
hypothetical protein PF1866	pf1866	18894055	-	44253,4	32	7	5	14	13	0,655	0,433	7	0,655	26 %
hypothetical protein PF1837	pf1837	18894020	-	26427,3	19	3	2	5	4	0,438	0,274	4	0,624	19 %
phosphoglycerate dehydrogenase	pf1394	18893507	-	33801,4	25	3	4	4	5	0,318	0,445	5	0,585	16 %
phospho-sugar mutase	pf0588	18892584	-	49601,1	36	7	5	11	10	0,565	0,377	7	0,565	19 %
flagellin	pf0337	18976709	SP III	28622,6	21	3	4	7	11	0,389	0,551	4	0,551	25 %
carboxypeptidase 1	pf0456	18892427	-	59005,7	43	5	6	8	11	0,307	0,379	8	0,535	12 %
alpha-glucan phosphorylase	pf1535	18893671	-	97643,7	71	13	9	30	17	0,524	0,339	13	0,524	20 %
Alpha-amylase	pf0272	1351936	-	76261,1	55	7	5	12	8	0,341	0,233	10	0,520	15 %
perplasmic sugar binding protein	pf0119	18892036	SP II	61223,5	44	4	8	7	15	0,233	0,520	8	0,520	20 %
hypothetical protein PF1111	pf1111	18893184	-	40301,7	29	2	4	3	7	0,172	0,374	5	0,487	13 %
phosphoenolpyruvate synthase (pyruvate, water dikinase)	pf0043	18891945	-	90427,9	65	11	5	21	11	0,476	0,194	11	0,476	17 %
acetyl coenzyme A synthetase (ADP forming)	pf1540	18893678	-	49933,2	36	6	2	13	4	0,468	0,136	6	0,468	17 %
putative sugar transport ATP-hydrolyzing	pf1933	18894131	-	41308,0	30	3	3	3	3	0,259	0,259	5	0,468	13 %
flavoprotein	pf0751	18892770	-	47371,7	34	4	3	9	3	0,311	0,225	5	0,403	14 %
hypothetical protein PF1304	pf1304	18893406	SP III	93671,7	68	6	9	13	22	0,225	0,356	9	0,356	15 %
aspartate/serine transaminase	pf1472	18893598	-	42898,5	31	4	2	5	5	0,346	0,160	4	0,346	14 %
putative sugar transport inner membrane protein (malg-like)	pf1936	18894134	-	45126,4	33	3	4	3	7	0,233	0,322	4	0,322	12 %
phosphonopyruvate decarboxylase bcpc	pf1959	18894161	-	45285,4	33	2	4	3	5	0,150	0,322	4	0,322	11 %
prolyl endopeptidase	pf0825	18892854	-	70779,6	51	3	6	6	10	0,145	0,311	6	0,311	11 %
IAA-amino acid hydrolase	pf0597	18976969	-	48672,3	35	4	1	9	1	0,301	0,068	4	0,301	10 %
tungsten-containing formaldehyde ferredoxin oxidoreductase wor4	pf1961	18894163	-	69324,7	50	3	4	5	5	0,148	0,202	5	0,259	8 %
phosphoenolpyruvate carboxykinase (gtp)	pf0289	18892234	-	72619,3	53	3	2	4	2	0,139	0,091	4	0,190	6 %
formaldehyde:ferredoxin oxidoreductase wor5	pf1480	18893608	-	64853,6	47	3	1	6	1	0,158	0,050	3	0,158	5 %
oligopeptide ABC transporter (oligopeptide-binding protein)	pf1209	18893298	SP I	71561,6	52	2	2	4	4	0,093	0,093	3	0,142	4 %

[a]A recent study has shown that pf1109 and pf1110 represent a single ORF, encoding a starch-binding protein (Comfort et al. 2008). In the text we are using the term PF1109 for this gene product. In the Table, the proteins are listed separately, corresponding to the original annotation in the NCBI database.

[b]Maximum number of unique peptides assuming 100 % efficient cleavage by trypsin

[c]Values under "Unweighed spectrum counts" are colour-coded (green, highest; yellow, lowest)

[d]emPAI values are colour-coded (red, highest; yellow, lowest)
