# Supplementary material for: Experimental and computational analysis of the secretome of the hyperthermophilic archaeon Pyrococcus furiosus
Source: Extremophiles. 2013 Aug 27;17(6):921–30. doi: 10.1007/s00792-013-0574-0 (PMC3824201; doi:10.1007/s00792-013-0574-0)
Supplement: Supplementary file 2 — Supplementary material 2 (PDF 81 kb) [file 792_2013_574_MOESM2_ESM.pdf]

Table S2: Prediction of secreted proteins in *Pyrococcus furiosus*. The predictions were made using PRED-SIGNAL and LipoP, with some manual adjustments.

Accession No.[a]	Gene	Gene product[b]	N-region[c]	H-region[d]	C-region[e]	Position +1 to +10
**Proteins predicted to be secreted by PRED-SIGNAL, with a positively charged N-terminus and a putative SPI cleavage site**						
18976406	pf0034	hypothetical protein PF0034	MLRR	IAAIILGILITVGM	AKA	DTKATPTPPN
18976416	pf0044	hypothetical protein PF0044	MKDKIR	LLAVVIPLIIGILTFPPNSTLTY	DGA	LYINIARSIA
18976516	pf0144	hypothetical protein PF0144	MKVR	VSYGTAIAMGLIKAKMLSSP	TTA	YLMTYHNGRC
18976520	pf0148	hypothetical protein PF0148	MRRD	QFFAGIIGPIIAYLGI	LSA	IYVQRSWWRL
18976546	pf0174	preprotein translocase subunit SecD	MKWRRILLNFR	VIVLIFFLLISITALA	TRG	LTFGLDISGG
18892121	pf0190	hypothetical protein PF0190	MRKK	LVGILTILVALGMLVSPLLKP	VAA	EDQKVLKIAM
18976633	pf0261	hypothetical protein PF0261	MKK	TSLMLIALIGLSLLSP	VIA	QEDIARRNYT
18976643	pf0271	hypothetical protein PF0271	MDKK	ITAVAIIFIVLL	VLA	MRTAKMPPAT
18976649	pf0277	hypothetical protein PF0277	MKK	ILPFLILLFSVGNVL	AMS	IELGIDEVLI
18976675	pf0303	hypothetical protein PF0303	MK	SLFLLVLGNTEVSLIPGI	SVA	GATPELTKYT
18976696	pf0324	hypothetical protein PF0324	MEKVNK	FGMAMLALLAAFGLI	LGA	GANFRDYNAS
18976697	pf0325	hypothetical protein PF0325	MNK	LFGIALFLAGLMLAV	GTG	ANFRYFEADR
33359468	pf0326	hypothetical protein PF0326	MLKMRLRASD	VLMLLVLGGVVFPS	VAG	YVMGRPIFVS
18976699	pf0327	hypothetical protein PF0327	MKK	FIGILLIALALVLVI	SSS	ANFREYQATR
18976700	pf0328	hypothetical protein PF0328	MKRIDKR	VFYLSVGAFIVLTMLS	TIA	YLAEPTYKRS
18976729	pf0357	dipeptide-binding protein	MKK	YLATVLVAALVLSNLGVV	LAA	EELPREETLY
18976745	pf0373	ABC transporter periplasmic binding protein	MRK	AVVLLIAITLFVRP	VIS	QEREKPLVVT
18976773	pf0401	methyltransferase	MKIKNNK	IILGILVVGVIVILA	STA	RGSFLDIADS
18976778	pf0406	hypothetical protein PF0406	MRR	AIYFSLIAIGIFFMISPLTVPLFTNTTPFSIFNPSPMGAS	SFA	KVLFQGGEIY
18976781	pf0409	hypothetical protein PF0409	MNLLK	PQIAGGIVAITTGILTSLAG	VFA	GNVQIINIGL
18976787	pf0415	dehydrogenase	MKK	FISLIVLFLLLPF	VQA	WEGKICENIE
18976792	pf0420	hypothetical protein PF0420	MRR	FIAVILLGLIGSLLI	SSA	KPILISIETP
18976808	pf0436	hypothetical protein PF0436	MKR	GLFLTLFFLLILGIAGHWAWN	SFG	SKPNSMQVTN
33359480	pf0437.1n	hypothetical protein PF0437.1n	MKK	LIAYMLVISLLAAGFYMENIGYTRFH	VAA	SSSVSWTEVL
18976815	pf0443	membrane transport protein	MGNIRRR	VSIVLLILMAAF	LMA	DQNLLPPNYQ
18976825	pf0453	hypothetical protein PF0453	MRR	WIISLILITLIMPI	VSA	QLLEFEPEGK
2183106	pf0477	alpha amylase	MNIKK	LTPLLTLLLFFIVLASPV	SAA	KYLELEEGGV
18976875	pf0503	iron ABC transporter	MKK	VITALILASLASVILGV	VFG	SVRIPLNDVV
18976880	pf0508	integral membrane glycosyltransferase	MKKR	TTIALLGILTLYVALRVPPLFWMD	EFS	DYDEGTYLLI
18976887	pf0515	hypothetical protein PF0515	MKK	SLIVFFAILVVMGILYGS	TTG	RVRSAFSSSI
18976892	pf0520	hypothetical protein PF0520	MGRWK	TVILDTLVMTAGFGTLTFM	SVA	KPDIIGHFNI
18976902	pf0530	hypothetical protein PF0530	MKR	QIILGFLVIILALAITLP	LAS	SNPDGLEATM
18976910	pf0538	hypothetical protein PF0538	MSRRKFLVR	VLLLIFLSGNMVNALNY	LAS	NEDSITRILG
18976953	pf0581	hypothetical protein PF0581	MKLR	TIAIILALVTLAS	VSS	IAMENFMDIE
18976959	pf0587	hypothetical protein PF0587	MIRH	AVVFFFLLLIGAFVF	SRA	IKTVEVQVLV
33359489	pf0600	hypothetical protein PF0600	MVEGMRR	VIASLGILLLLSPL	ALA	CFFPEDNYAL
18976993	pf0621	hypothetical protein PF0621	MKK	AIILLLLLLLPV	VSA	QKVKVVVYDD
18977022	pf0650	hypothetical protein PF0650	MFFGVKKYEKIR	ILGAGIIIIMLFLVAS	IVS	DTRKEQVQGS
18977023	pf0651	hypothetical protein PF0651	MVIPIKWK	LFGVLLLGLMALVSIPK	VQG	NDVGGVMTFT
18977053	pf0681	hypothetical protein PF0681	MQKK	ILGFIVSILVLGMTIEVV	SSA	KVPNEHKNVL
18977077	pf0705	cytochrome c-type biogenesis protein	MRR	ALLTFIVLLLFAGL	VTG	REVSYGGLKF
18977096	pf0724	hypothetical protein PF0724	MKLK	ALFLLLALGSLIVPR	IEA	EEFESSKVHF
18977178	pf0806	hypothetical protein PF0806	MR	VIYAIMLIFGLGFLILPISIPVFL	SNA	EFSILNNKWN
18977179	pf0807	hypothetical protein PF0807	MKR	VSLLLIFLLITP	VLA	EPRQIPLKEV
18977186	pf0814	hypothetical protein PF0814	MKK	LITLLLLPLLLTFALFDENKTF	IFA	RNVTFIGKGS
18977220	pf0848	hypothetical protein PF0848	MKK	NMIGFGILLFLFIFMA	YKA	LESPEPQKVI
18977226	pf0854	endo-1,4-beta-glucanase b	MSKKK	FVIVSILTILL	VQA	IYFVEKYHTS
18977259	pf0887	hypothetical protein PF0887	MPR	LVIPILALLIFSAPM	ASA	VYSVKQGGFL
18977261	pf0889	hypothetical protein PF0889	MRK	YIIVGILVLFLSSTT	AYS	TLGTLLGYVD
18977321	pf0949	hypothetical protein PF0949	MKRK	GLALGMVFVVFLMIFT	SYA	GITTGEFWND
18977322	pf0950	hypothetical protein PF0950	MKEARK	LILPLAILLITIPM	AVA	EGWAKTYKIE
18977327	pf0955	hypothetical protein PF0955	MQIFVVVVLGLR	WVIVLLLGFVVLGSFGSAGN	DKA	CQPVKYWVFE
18977334	pf0962	hypothetical protein PF0962	MLK	NVFALVVCIIILSGTVSA	TKG	IINPTYIDPG
18977367	pf0995	hypothetical protein PF0995	MRK	GILIFTLISFLLLNTP	VLA	QEPLNNIDLQ
18977372	pf1000	oligopeptide transport system permease protein appc	MKRK	IGVVLLVLLGLFVVLSNLS	VSS	DEIANWVNTN
18977373	pf1001	oligopeptide transport system permease	MKFPTKTLMK	VAAVYACVFLAIIL	VAG	ITANRLTWEY
18977376	pf1004	alkaline phosphatase	MKK	ITISSLLLLLLISTNL	NLA	YDSQESGIKN
18977385	pf1013	hypothetical protein PF1013	MRR	VSVIILTIAIFLAIGLAVDY	ITS	TYGSINYEWK
18977401	pf1029	hypothetical protein PF1029	MKK	MLLLLMVLFFLISSP	VSA	SYITKNFTLI
18977452	pf1080	hypothetical protein PF1080	MKVKLR	SLIAVLLGLLIIGANTE	SVA	ARPVDDTKPK
18977478	pf1106	hypothetical protein PF1106	MR	SSIVFGALGAVILILVSLTVKPFFSSFFFAF	VTA	YVLSPLHTKL
18893182	pf1109	hypothetical protein PF1109	MRR	NAQVFAMVLLLVLSGIPK	ALA	LYTPTPFSID
161484709	pf1154	NAD-dependent deacetylase	MLGEVSKILAK	SSMAIAFTGAG	ISA	ESGIPTFRGK
18977553	pf1181	hypothetical protein PF1181	MNKWIGR	FLIFIVITSLVWSS	VSA	QWMLSDDGIT
18977558	pf1186	NADH oxidase	MEKKK	VVIIGGGAAGM	SAA	SRVKRLRPEW
18893298	pf1209	oligopeptide ABC transporter (oligopeptide-binding protein)	MKR	LVGVLIGAFVIFGVFGQV	VAA	QEQELPREET
18977591	pf1219	hypothetical protein PF1219	MHSH	GLVVLPISSSIGALVF	ERG	RYRVIAKPGE
18977606	pf1234	chitinase	MKTR	MLGIVLAWLVVLSLVSPTISLFYP	VSA	QQTVQLDGYA
18977622	pf1250	hypothetical protein PF1250	MKR	FFAFAMVLLFLLSALPV	FGA	QEYVKKVSVF
18977678	pf1306	hypothetical protein PF1306	MVGALRGQVFSMD	ALVSMVIVVMIIGTVS	ATS	ESIKNEIVSL
18977705	pf1333	phosphomethylpyrimidine kinase	MKRLVK	TALTIAGSDSGGGAGIEADLKTFTAFGVHGLVAITS	VTA	QNTQAVTAIH
18977768	pf1396	2-dehydropantoate 2-reductase	MK	IYVLGAGAIGSLFGGLLF	AAG	EDVVLIGRKP
18893514	pf1399	putative ATPase, vanadate-sensitive	MKVKK	IAALAVGAAVAGATLGF	ASA	QGEVPEIPKD
18977772	pf1400	hypothetical protein PF1400	MRK	YIAGIFLITIILASIGIT	AYG	YAKFNSILIS
18977799	pf1427	hypothetical protein PF1427	MKR	ALGFLSLLVIFASL	LVA	LSPEYGIKFG
18977810	pf1438	hypothetical protein PF1438	MRGK	ILSFLIVLTLLALP	TTA	QCPSLGRTVV
18977835	pf1463	hypothetical protein PF1463	MNNIKVYLDRKH	FVVMLLMFLAVSTTT	TKA	GSLEPYLLDR
18977836	pf1464	hypothetical protein PF1464	MKK	TVFLLFIFLMLTPN	TLA	SESIPLKWRV
18977874	pf1502	hypothetical protein PF1502	MKK	VALFFFIIVISLFMPP	TTA	QPTLLISISP
18977876	pf1504	large helicase-like protein	MHK	YFFPLPATKSTFLLPADL	TTA	NPCFSKSLIN
18893637	pf1505	hypothetical protein PF1505	MKK	ASILLIIMLIASGLTIFN	PKA	LGLEKYSTLT
18977878	pf1506	hypothetical protein PF1506	MRK	LYVLSLLLLIILGSSA	VSA	SILEYSSKIT
18977885	pf1513	hypothetical protein PF1513	MMISKR	IMLMILGVVILTPTVS	ALS	ISCPSEVLYD
18977892	pf1520	ABC transporter	MKR	LLPLSLLFLGVFFYLPIFLIVKEGID	VKA	LVDVVTDSYH
18977897	pf1525	hypothetical protein PF1525	MR	GVFISYFFLVFFVGLL	SLG	YSTTIVQGTV
18977905	pf1533	nodulation protein nfeD	MTMKK	AIMILTIFTLLLFAQG	VKG	RDSNVVYVAQ
18977993	pf1621	hypothetical protein PF1621	MKIYK	VISVLLILLVL	PLA	EATELPWSEI
18978041	pf1669	hypothetical protein PF1669	MKK	VFLGVIIITLLLSP	VKA	GEYLYVYKPT
18978087	pf1715	pyrroline-5-carboxylate reductase	MK	VAVIGAGTIGSAV	AKA	LAESNYEVIA
18978113	pf1741	trehalose/maltose transport inner membrane protein	MREEVLKR	ILLIIGAILMAIICLFPFIWMIVV	SFA	EDPTFLGSPL
18978120	pf1748	sulfate transport system permease, ABC transporter	MRMKWR	NFIVFLPLVFLFMFFYFP	LMS	ILKEGLWDNG
18978134	pf1762	hypothetical protein PF1762	MKFK	PLTLLLLSFIITSTIPGE	VAS	QVHGIVILVS
18978142	pf1770	2-oxoglutarate ferredoxin oxidoreductase subunit gamma	MQIR	FAGIGGQGVVLAGVILGE	AAA	IEGLRVLQTQ
18978145	pf1773	2-oxoglutarate ferredoxin oxidoreductase subunit gamma	MRKE	ILLGGFGGQGIILASVILGR	AAA	VYEGLYAVQT
18978160	pf1788	NDP-sugar dehydratase or epimerase	MKNK	LVVVTGGAGFIGSHI	AEA	LVEENEVIVI
161484706	pf1839	CTP synthetase	MTK	YIFVTGGVVSGLGKG	ITS	ASIGLLMKAR
18978213	pf1841	hypothetical protein PF1841	MREIGKK	YILAISFIFLIGISI	SLA	EYYSLPMAVA
18978215	pf1843	chromosome segregation protein smc	MFFTR	NSASFSTSLISIIPV	INA	IVNNSIPRTL
18978277	pf1905	protease	MLGLR	WVIVLLLGFVVLGSFGSAGN	DKA	CQPVKYWVFE
18978278	pf1906	adenosylmethionine-8-amino-7-oxononanoate aminotransferase	MKRIFK	VTFMFFILLSPEL	TIA	YTTVNPLWSL
18978279	pf1907	hypothetical protein PF1907	MKK	LSALFVVLLVIGTVAQI	VAA	QTADSREVAP
18978280	pf1908	hypothetical protein PF1908	MRAKK	ISIFIFLFLIGASY	IYS	HVPNESVFLV
75993212	pf1934/1935	amylopullulanase	MSRK	LSLLLVFLIFGSMLGANNI	VKA	EEPKPLNVII
18978309	pf1937	malf-like sugar transport inner membrane protein	MKK	TTVAALMLIIPGIAAFLFFNLWPI	VYS	IYLAFTNAKL
18978348	pf1976	L-aspartate oxidase	MK	VAVVGSGLAGLTA	AMS	LAKKGIEVTV
18978355	pf1983	hypothetical protein PF1983	MVRR	SIVFFVISLLLGGLVNPSI	VQA	STQDPYEEFW
18978377	pf2005	glycerol-3-phosphate dehydrogenase	MIK	VAIIGAGITGASIAR	VLS	RYENLEVHLI
**Proteins predicted to be secreted by PRED-SIGNAL, <u>without</u> a positively charged N-terminus**						
18976412	pf0040	type II phosphatidic acid phosphatase	MLAI	LTAILLTIFALQVLGAFNG	INS	LIDSSINLPT
18976421	pf0049	cytosine-specific DNA-methyltransferase	MPSVID	LFAGAGGFSLGFKLAGFKI	ISA	VENFKPKAKT
18976477	pf0105	hypothetical protein PF0105	MRQD	VVYLLMFFVFLSLAI	SLS	NPPPKDIIEA
18976478	pf0106	hypothetical protein PF0106	MRE	LLQLIAGVGFGTLTGLTPGLH	VNS	LSRLSLPIPT
18976489	pf0117	sugar transport protein	MKDVETRSKSSE	VVLILGLFLATLPLLVGFS	LLA	LSSFSTQMVT
18976511	pf0139	indolepyruvate oxidoreductase subunit B	ME	FNIIIAGVGGQGGLTLSRIIG	SAA	MVEGYRVRIG
18976550	pf0178	V-type ATP synthase subunit K	MD	PIVYVALGMALGAGIAGAASSFGVGIA	GAA	AAGAVAEDER
18976564	pf0192	oligopeptide transporter permease appc	MRWVDLKESLSDFWFEFRREK	TGIAGLALLIFWIVVA	VSA	PYVTEPNIPD
18976705	pf0333	flagella-related protein g	MAAGEPASE	LILFIVAVIVASTVAG	ALA	YVTTDIANGM
18976722	pf0350	cation antiporter	MIAL	VLASIATTLPEVT	VSA	ISSFKGESSI
33359479	pf0432.3n	hypothetical protein PF0432.3n	MIIT	IIIIAIIILTISAT	AYA	KVAFGECSQV
18976856	pf0484	GTPase	MIIA	FLGTAGSGKTTLTG	AFG	RYLEENYRVA
18976995	pf0623	hypothetical protein PF0623	MGAAER	IAFAVLSILIVLVAPFVR	VSS	EYYDETVTTT
18977069	pf0697	multidrug resistance protein	MMPI	IALYAISLGASLPQAGI	IAA	LYSIAAIPAS
18977070	pf0698	hypothetical protein PF0698	MALA	IAIGSATAGVLTARLGYRVSFA	ILA	LIIGLSSLIS
18977260	pf0888	hypothetical protein PF0888	MAKE	LMLLLVPISFLILFFP	AVS	ADCSPHEVYS
18977392	pf1020	phosphate transport protein pitb	MQLGFAMLAD	PILLITILLGFAMAWAIGANDAANSMSTAVG	AGA	ITPRQAVIIA
18977447	pf1075	hypothetical protein PF1075	MVFL	IVPILTILGLSIHEAIGT	SLA	CIAISSFSSA
18977467	pf1095	hypothetical protein PF1095	MNLS	AIFASLLGLSLSLF	TTG	VYRYYSKPKI
18977611	pf1239	hypothetical protein PF1239	MTLT	LIPFLMSIFIIGFATS	VLG	EEIPKQVILF
33359561	pf1357.1n	hypothetical protein PF1357.1n	MSE	VIYTIGAVLFGGGIG	NTA	YYEVEGLHRN
18977796	pf1424	monovalent cation/H+ antiporter subunit F	MIFF	YATLLIGIAGIITFI	RLA	LGPTVPDRVV
18977802	pf1430	monovalent cation/H+ antiporter subunit D	MTWL	PFIIIIPLLGAFSMPIVSL	LKG	KAKEIWASMI
18977895	pf1523	hypothetical protein PF1523	MTII	LSSLFLAGTLGVITFLASHPLEMTFLSFGV	SKG	EEETKTCAVN
18977906	pf1534	stomatin	MATP	IGFFTAAIILLFILIF	LSS	AIKIVKEYER

18978070	pf1698	ribose ABC transporter	MLED	VFVLLSNTLFSMVPL	VLA	GVGEVITERS
18978129	pf1757	hypothetical protein PF1757	MNPA	PFLLLGGALAWLTLYLLGGRK	VKA	GEFILGAIIF
18978217	pf1845	murein hydrolase	MNTF	GIFLTISAYTFF	SWA	YSKKKTPILN
18978295	pf1923	hypothetical protein PF1923	MFAL	ISFILVVVLSIIIVRIG	AIA	LEMTGLSRDV
18978356	pf1984	hypothetical protein PF1984	MVGS	VYTLFLYFSIILGVIVGVRTRSFHS	SLA	VLVFSSTFVA
18978404	pf2032	hypothetical protein PF2032	MSD	FGVLSLLPPLVAI	VLA	IWTKRVVFAL
**Putative lipoproteins**						
18976448	pf0076	endo-beta-1,3-glucanase[fh]	MKKE	ALLFLSLIFLVF	VSG	CIHHSTNQQL
18976452	pf0080	molybdate ABC transporter periplasmic substrate-binding protein[fh]	MREGGVMKKR	LLALIVAFAVL	TAG	CLGSESKEVT
18892036	pf0119	periplasmic sugar binding protein[fh]	MKHK	AVFLLVVLISGVL	ASG	CIGGETKETQ
18976832	pf0460	hypothetical protein PF0460[fh]	MRHLK	ILVVMMVLL	GLG	CLIKSPAQVR
18977283	pf0911	iron (III) ABC transporter[fh]	MKK	SLIALLIIFI	TLG	CINSPSTTQE
18977350	pf0978	hypothetical protein PF0978[gh]	MR	VPLLILLFLVL	TSG	CIAPSTPSFT
18977375	pf1003	phosphate-binding periplasmic protein[fh]	MKR	LATIFIIFALGLSLV	ASG	CISEGNASNV
18977472	pf1100	hypothetical protein PF1100[fh]	MKKINKK	ISILLTLLFFFPL	LLG	CINAPHTNTR
18977480	pf1108	alpha-dextrin endo-1, 6-alpha-glucosidase[fh]	MKR	MIMYLSTVLLIAV	VSG	CISEQTQTQT
33359548	pf1182.1n	hypothetical protein PF1182.1n[fh]	MRGGK	IIFALLMIMVVI	SAG	CTEKSTPTTT
18977670	pf1298	endonuclease[f]	MRAK	IAVVLILFLF	FSG	CTSRENELRG
18893525	pf1408	putative dipeptide binding protein[gh]	MKK	GLLAILLVGVMVLGTF	GSG	CIGGGTQTQT
18977890	pf1518	thiamin-binding periplasmic protein[fhi]	MNK	IVTGILLVLVLM	ASG	CITSPGEQEK
18893856	pf1695	hypothetical lipoprotein[fh]	MRK	VGITLSVVALVIMGF	VAG	CIGGTQTQGE
18978111	pf1739	trehalose/maltose binding protein[fh]	MNVKK	VLLGLFLVGVLGIAVV	ASG	CIGGQQTSTV
18978123	pf1751	solute binding lipoprotein[fh]	MKR	FAVILGLLLIAT	TLG	CITQSQTSTE
18893949	pf1774	iron (III) ABC transporter, ATP-binding protein[fh]	MKR	AIPVFLLIVLVW	ISG	CIGGGTSTIP
18202323	pf1938	maltotriose binding protein[gh]	MRR	ATYAFALLAILVLGVV	ASG	CIGGGTTTPT
18978339	pf1967	hypothetical protein PF1967[fh]	MNKK	ISMLLIVLLGIAVF	ASG	CIGGGGAKIV
18978345	pf1973	hypothetical protein PF1973[fh]	MRK	ISLLVLVVVSSII	VAG	CIGGGTSTAE
18978435	pf2063	aminopeptidase[f]	MMIGKK	VIVIFISFIIIL	PSG	CLRIPTEQKT
Proteins with Class III signal peptides[i]						
18976567	pf0195	hypothetical protein	MPLGRKNLKGQKMGLKKGIT	YPPLFVGLLLLLTSLILAYFSMY		
18892232	pf0287	pyrolysin[k]	MNKK	GLTVLFIAIMLLSVVPVHFVS		
18976709	pf0337	flagellin[k]	MKKGAI	GIGTLIVFIAMVLVAAVAAGVLI		
18976710	pf0338	flagellin[k]	MKKGAI	GIGTLIVFIAMVLVAAVAAGVII		
18976768	pf0396	hypothetical protein	MSISTSSSAPPK	GFLIVFGNFIFPKALFIVPFVVPG		
18976779	pf0407	hypothetical protein PF0407[k]	MASGGDAPVILTVSAFDTVNMKK	ALVILVVLITMISPIFAVE		
18976844	pf0472	hypothetical protein	MKRAQT	AIEYLLMLAAVLILVAIVLNVVV		
18976859	pf0487	autolysin-like protein[k]	MKKA	IVFMTVISFLLLIPPVLGE		
18893406	pf1304	hypothetical protein PF1304[k]	MRRG	FIINSTLLILIIPLLLLAATYAEI		
18977677	pf1305	hypothetical protein PF1305[k]	MRKG	FIFTLDALLALLLTTVVVAGTFSL		
18977881	pf1509	PF1509 hypothetical protein	MKKGQV	SLEFLFIFMIFTLILIYSI		
18977882	pf1510	PF1510 hypothetical protein	MKAQLSI	LSIDFIFAMTLLLIFSLGLMSFA		
18978042	pf1670	alkaline serine protease	MKGLKA	LILVILVLGLVVGSVAAAP		
18978092	pf1720	hypothetical protein	MRRGLS	LLGFIIGAVIGIIIGALILAGI		
18978133	pf1761	hypothetical protein PF1761[k]	MRGVLS	LLVVAMLVLSMGVVPGVTAATST		
18978437	pf2065	aminopeptidase[k]	MKRKA	LAVFLISFLVLSVGYILWSN		
33359466	pf0298.1n	hypothetical protein	MKA	LIVSLFRFLLYVPRVLFYIAGIIR		

[a]Accession number from NCBI database

[b]Data extracted from the NCBI database

[c]The N-region is defined as the N-terminal part from the first residue to the last positively charged residue. In predicted SPs without N-terminal charged residues, the n-region is defined as the first four residues

[d]The H-region is defined as starts after the last charged residue and ends at postion -4 upstream of the predicted cleavage site

[e]The C-region is defined as three residues upstream of the predicted cleavage site

[f]Lipoprotein signal peptides predicted by LipoP 1.0

[g]Lipoprotein signal peptides predicted manually

[h]Lipoprotein signal peptides predicted by PRED-SIGNAL

[i]Class III signal peptides predicted previously using FlaFind by Szabó et al. 2007

[k]Proteins also identified by PRED-SIGNAL
